# Acute Otitis Media during the First Two Years of Life in a Rural Community in Bangladesh: A Prospective Cohort Study

**Published:** 2007-12

**Authors:** Eliza Roy, Zahid Hasan, Fazlul Haque, A.K.M. Siddique, Richard Bradley Sack

**Affiliations:** 1 1CDDR,B, GPO Box 128, Dhaka 1000, Bangladesh; 2 Department of International Health, Johns Hopkins Bloomberg School of Public Health, 615 North Wolfe St., Baltimore, MD 21205, USA

**Keywords:** Cohort studies, infant, Child, Morbidity, Otitis media, Prospective studies, Bangladesh

## Abstract

The study investigated the burden of acute otitis media (AOM) during the first two years of life in a cohort of 252 newborns in rural Bangladesh using data collected on occurrences of AOM. Trained community health workers (CHWs) conducted household surveillance and picked up cases of AOM using the study algorithm. The incidence rate was 0.9 episodes per child-year observed. Forty-six percent (n=115) of the 252 subjects developed AOM: 36% (n=91) during the first year of life and 10% (n=24) during the second year of life (p<0.001). The age-specific incidence rates of AOM varied; peaks occurred in the 6-12-month age-group and the lowest in the first three months of life. In total, 20% (n=49) of the study subjects had single, 26% (n=66) recurrent, and 54% (n=137) no episode of AOM. Perforation with discharge developed in 85% (n=322) of 375 episodes. The duration of discharge from the ears was ≤6 weeks in 95% of the episodes, but in 5% of the episodes, discharge from the ears continued for >6 weeks. The incidence of AOM was higher in the monsoon season compared to the summer season (p<0.003). The study documented AOM as an important cause of morbidity among rural children up to two years of age in Bangladesh and should be addressed with strategies to overcome the burden of disease.

## INTRODUCTION

Acute otitis media (AOM) is a worldwide, commonly-diagnosed childhood disease of the ear. In the USA, infants average approximately 1.2 and 1.1 episodes in the first and the second year of life respectively (1,2). The prevalence of AOM is also high in developing countries and is an important cause of morbidity and mortality in children aged less than five years (under-five children) (3). Data of a survey in Bangladesh in the 1980s showed a high prevalence of chronic suppurative otitis media (CSOM) in rural, urban and kindergarten school children with prevalence of 43.2/1,000, 32.6/1,000, and 16.3/1,000 respectively (4). Similarly, a hospital-based study showed complications in 85% of subjects aged 5-50 years (5). Data on the burden of the disease in children aged less than two years are sparse from this country. This study highlights the epidemiology of the disease in children aged less than two years and focuses on its burden using data collected on AOM in a rural community in Bangladesh.

## MATERIALS AND METHODS

### Study area and study population

The study was conducted in 10 villages in Mirzapur, a rural subdistrict situated at a distance of 60 km northwest of Dhaka, the capital city of Bangladesh, during August 1993–September 1996 (6). At the time when the study was conducted, the number of people was 16,510 in 2,962 households in the study area. Males comprised 51.9% (n=8,565) and females 48.1% (n=7,945) of the population. The under-five children constituted 10.8% (n=1,551) of the total population. About 40% (n=3,181) of the 7,945 females were of childbearing age, and 77.8% (n=2,475) of them were married. The study subjects were babies born during October 1993–September 1994 who were recruited in a larger study on aetiologies of acute lower respiratory tract infections and of diarrhoea.

### Field team and training of staff

The field team comprised a study physician, a research nurse, and 12 community health workers (6). The study team received two weeks of training at the Kumudini Hospital, a 500-bed general hospital in the study area, in the assessment of clinical findings and diagnosis of AOM and documentation of outcomes. To ensure the reproducibility of results, they were given refreshers’ training every two months throughout the study period. The study algorithm used in this community-based study focused on confirmation and referral of cases from the field level. Special emphasis was given on teaching the Community Health Workers (CHWs) in how to take ear swabs from babies. The same group of the study staff members was also trained to collect information on acute respiratory tract infections (ARTIs) and diarrhoea using the guidelines of the World Health Organization (WHO), for their diagnosis at the community level (7,8).

### Surveillance activities

The CHWs conducted a door-to-door census in the villages and identified married women likely to become pregnant and deliver babies, during August 1993–September 1994, to participate in the study. A system was developed to report the birth of any baby in the study area either by the families or by the CHWs in the study area. A physician and a staff nurse visited the newborn as soon as the CHW/family reported any delivery. Informed consent was obtained from a parent or guardian for inclusion of each child in the study. Birthweight was assessed with a Salter spring scale with 100-g precision, and detailed information on childbirth was collected. Each child was given a unique identification number at the time of enrollment into the study (6).

The CHWs visited the homes of the participants every fourth day and conducted interviews on the preceding three days to collect information on the occurrences of AOM. They also collected and documented information on diarrhoea, respiratory tract infections (RTIs) in accordance with the standard algorithms of WHO, and morbidity from other illnesses using the study algorithm that was set up.

The surveillance continued until each child reached two years of age. Baseline information on household characteristics and other sociodemographic characteristics was collected from families of enrolled babies. This included data on monthly income and expenditure, housing structure, number of rooms, ownership of a tubewell, maternal age and education, paternal education, smoking habits, and child characteristics, such as sex, birth-weight, and birth-order. Information on breastfeeding, duration of breastfeeding and dietary habits was also recorded. The days the child was absent from the field station, dates of out-migration, and dates of deaths were documented. To ensure the quality of data, monthly meetings, supervision and reinterview of a random sample of the study children were conducted during the entire surveillance period.

The Research Review Committee and Ethical Review Committee of ICDDR,B approved the research protocol.

### Criteria for assessment of AOM, study definitions, and measurement of outcomes

We aimed to pick up all cases of AOM from the community by setting up an algorithm. Trained CHWs and the study physician were involved in the diagnosis. During a house-visit, a clinical suspicion of AOM was made by the CHW when a mother reported that her child had been suffering from one or more sign(s) of acute illness that included (a) earache, (b) fever, (c) irritability/lethargy, (d) vomiting, or (e) diarrhoea. The ear(s) of every child with clinical suspicion was/were examined, irrespective of the presence of discharge. In the community, during the household surveillance, the CHWs made a clinical case diagnosis of OM only after verification of the presence of pus/discharge by taking ear swabs. In the absence of pus/discharge, the case was referred to the study physician. The study physician performed an otoscopic examination of the referred case(s) and made a diagnosis of otitis media by correlating clinical and otoscopic findings. The otoscopic findings that aided the physician in this rural setting were a bulged tympanic membrane showing discoloration or the presence of discharge/pus.

The primary clinical outcomes of the study were based on the number of episodes, condition of the ear(s), and time required for resolution (not meeting the definitions for CHWs and the physician). An episode was defined as (a) single episode if only one episode occurred during the two-year follow-up, (b) recurrent otitis when two or more episodes of AOM in the same child within six months (9), (c) acute otitis with perforation if discharge was present in the ear on swabbing, (d) AOM without perforation if the tympanic membranes were intact on otoscopic examination, and (e) chronic supportive otitis media (CSOM) if discharge continued beyond two weeks (10) with no resolution on follow-up.

Each child with a diagnosis of otitis media was closely monitored for resolution of an episode. In this community-based study, it was not possible to determine resolution after doing an otoscopic examination. The ear(s) of the child was/were swabbed on every visit to confirm if it was free of discharge. The study algorithm defined resolution as subsidence of symptoms in a child without perforation or absence of any discharge/pus from the ear(s) in a child with perforation. The interval between the onset as reported by the mother/caretaker and resolution was used for determining the duration of an episode. A 21-day disease-free interval was required to define an episode as a new episode (9), otherwise it was counted as continuation of the same episode.

Upper respiratory tract infections (URTIs) are common in children with AOM. During an episode of the disease, the CHWs collected information on upper respiratory illness defined as cough, running nose, or sore throat using the guidelines of WHO (7).

### Statistical analysis

Data were entered using the FoxPro program, and statistical analysis was done using the SPSS software (version 10) for Windows. Incidence density rates were calculated using the number of either single episode or recurrent episodes (numerator) experienced by the 252 newborns divided by number of days of observation starting from birth up to two years (denominator) minus days with illness and days absent. The children with first episodes at three-monthly age intervals and the number of children experiencing single and recurrent episodes were calculated. Both age-specific incidence rates and prevalence were calculated. Analysis was performed for the first and the second years of life separately and for the two years combined.

Descriptive statistics, such as frequency, cumulative frequency, percentages, means, and standard deviations were used for describing the study sample. Statistical comparisons were made using the Student's t-test and χ^2^ test as appropriate. A p value of <0.05 was considered significant.

## RESULTS

In total, 288 children were born from August 1993 to September1994. Of these, 252 were kept under surveillance for two years, and 36 were not included in the analysis. Out-migration (n=11), congenital abnormalities (n=5), and deaths in the perinatal (n=7), early neonatal (n=9), and postneonatal (n=4) periods were the reasons for exclusion. Males comprised 56% and females 44% of the birth cohort (n=252). About 34% of the newborns had a birthweight of less than 2.5 kg. The cohort children came from families with a median household income of US$ 60 per month. The mean maternal age was 25 years, and 70% of them had less than five years of education.

During the surveillance period, 151,597 child-days of observation were made, and 375 episodes were documented giving an overall incidence rate of 0.9 episodes per child-year of observation. The age-specific incidence rate of AOM varied with the peak incidence in study subjects aged 6-12 months. The lowest incidence of AOM occurred in the first three-months of life. The prevalence was 6-11 days per 100 days observed in different age-groups (Table).

**Table:** Incidence and prevalence of AOM by age of study children (n=252), Mirzapur, Tangail, August 1993–September 1996

Age-group (months)	Child-days observed	No. of episodes	Incidence∗	Prevalence†

0-3	18,404	18	0.4	11
3-6	19,350	47	0.9	8
6-9	19,036	73	1.4	8
9-12	18,594	72	1.4	7
0-12	75,384	210	1.0	8
12-15	19,351	47	0.9	6
15-18	18,913	47	0.9	7
18-21	19,055	37	0.7	6
21-24	18,894	34	0.7	6
12-24	76,213	165	0.8	6

∗Per child-year observed incidence rate=No. of new episodes of AOM/total days observed ∗ 365

†Per 100 days observed prevalence rate=No. of days with AOM/total days observed ∗ 100

AOM=Acute otitis media

**Fig. 1:**
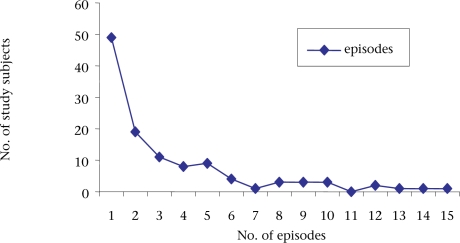
Study subjects experiencing single and recurrent episodes (n=115)

In total, 46% (n=115) of the 252 study subjects developed AOM during the two-year follow-up: 20% (n=49) had only one episode, and 26% (n=66) had recurrent episodes (Fig. 1). The mean (±SD) number of episodes among those experiencing AOM was 3.1±1. Of the 91 subjects experiencing AOM in the first year of life, 30 (33%) had single and 61 (67%) had recurrent episodes. In the second year of life, 21% (5/24) had single, and 79% (19/24) had recurrent episodes. The overall number of recurrent episodes was higher (p<0.02) than single episodes. About 44% (64/143) of male and 50% (55/109) of female subjects developed the disease. The mean (±SD) number of episodes in males was 1.65±3.1, and in females, it was 1.28±1.9 during the follow-up period. Of 375 episodes of the disease documented during the surveillance, two-thirds (n=236) occurred in male babies.

Forty-nine children had single, and 66 had recurrent episodes∗ among the cohort children (n=252) followed from birth to 2 years in Mirzapur, Tangail, Bangladesh, August 1993–September 1996 (∗p<0.02)

**Fig. 2:**
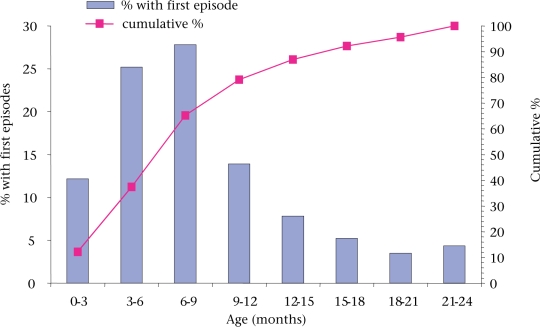
Distribution at 3-monthly age intervals of study children (115/252) with first episodes of AOM during follow-up from birth up to 2 years of age in rural Mirzapur, Tangail, Bangladesh, August 1993–September 1996

The first episodes of AOM in the study children are shown at three-monthly age intervals in Figure 2. The incidence was highest in the 6-9-month age-group, followed by the 3-6-month age-group. The number of subjects (91/252) who experienced their first attack of AOM in the first year was significantly higher than those (24/252) in the second year of life (p<0.0001).

In this cohort, 60% of the subjects were exclusively breastfed for three months. The incidence of AOM in these exclusively breastfed subjects was 0.8 episodes per child-year of observation. In the remaining 40% of the subjects who were not exclusively breastfed for three months, the incidence rate was 1.04 episodes per child-year of observation.

**Fig. 3:**
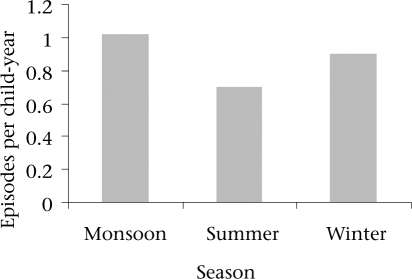
Seasonal distribution of incidence rates of acute otitis media infections among cohort children (n=252), Mirzapur, Tangail, Bangladesh, August 1993–September 1996

Perforation with discharge from the ears was common and was associated in 85% (n=322) of the 375 episodes. Of these, 218 and 104 episodes were, respectively, associated with unilateral and bilateral discharging ears. The duration of ear discharge was ≤6 weeks in 95% of all the episodes documented but in 5% it continued for >6 weeks. In total, 7.5% (19/252) subjects had no resolution and continued as CSOM. Resolution is common in AOM of viral aetiology. The incidence of acute URTIs in this population was 7.5 episodes (2,469/1,19,468) per child-year of observation (unpublished data). Among these URTI episodes, 11% (281/2,469) were associated with clinical suspicion of ear symptoms relating to AOM. Of 115 children experiencing AOM, 73 (63%) had symptoms of URTIs meeting the WHO criteria of diagnosis. The incidence rates of AOM in the summer (March-June), monsoon (July-October), and winter (November-February) are shown in Figure 3. Incidence rate in the monsoon season was higher than that in the summer season (p<0.003).

## DISCUSSION

The incidence of AOM in the study population indicates that it is a frequent cause of morbidity in children during the first two years of life in rural Bangladesh. The findings corroborate the high prevalence shown in a survey report on otitis media in Bangladesh (4). In the USA, the disease continues to present as a common childhood disease with rising trends in the prevalence. The reports of the Third National Health and Nutrition Examination Survey (NHANES) showed a 11% increase in the prevalence of early onset OM (<12 months of age) and 18% increase in the prevalence of repeated episodes (≥3 episodes) among children aged less than six years (1). Data from other developing countries also estimated combined loss of life from premature deaths and disability from complications of AOM to be high in under-five children (3). No follow-up studies on long-term outcomes of AOM have been done in Bangladesh; however, a survey showed that CSOM is common (4). Results of another hospital-based study showed that complications with CSOM were high (5).

The findings that 36% of the cohort children were affected in the first year of age are similar to those seen in Alaskan Eskimo children in the 1980s when the incidence was highest in North America (11). However, it was 25% lower than those reported in the Greater Boston studies (9) where 45% of children were affected, but rates were higher than in a Swedish study (12) where only 21% had experienced episodes of AOM by one year of age. The higher number of episodes of AOM experienced in the first year than in the second year of life (p<0.001) is similar to the Greater Boston cohort study where incidence rate decreased from 1.2 to 0.4 in the second year (9).

The number of children with first attacks was lower in the second year but the recurrent episodes continued to be high. Two-thirds (60/91) and four-fifths (19/24) of the study children with first episode in the first and the second year of life respectively had recurrent episodes. Recurrence rate is high in children with the first episode of the disease that starts early in life (1,9,11,13).

When examined in smaller age intervals, the lowest age-specific incidence rate was observed in the first three months of life, and the highest incidence rates occurred in the second half of the first year of life (Table). This high incidence corroborates with the Greater Boston studies where the highest incidence rates were observed in a similar age-group (9). The effect of breastfeeding in lowering rates of AOM has been documented in other studies in Denmark (14,15) and New York (19). In our study, 60% of the babies were exclusively breastfed for three months. Those subjects who were exclusively breastfed had a lower incidence rate of 0.8 episodes per child-year of observation compared to 40% of the subjects who were not exclusively breastfed who developed 1.02 episodes. Maternal transfer of IgG_2_ antibodies to babies through breastmilk has a role in combating infections (15,16).

In our study, 63% of the AOM cases had URTI as an association. URTI in association with AOM is well-documented (18,19,20,21). We presume that it could be important in the aetiopatogenesis of AOM in our population. The frequency of AOM in children with respiratory infections usually ranges from 10% to 50% (18,19). A study in an urban paediatric outpatient population in Finland reported symptoms of URTI in 94% of subjects with new episodes of AOM. Respiratory viruses were detected in cultures of the nasopharynx in 42% of referenced study subjects (22). In our study, the high incidence rates were observed in the monsoon season; this correlates with seasonal peaks of URTI seen in Bangladesh (23). We also presume that the association of URTI with AOM in the study subjects could be related to prolonged symptom but resolution in six weeks in 95% (356/375) of episodes. Several studies have reported an association of AOM with viral URTIs and high resolution rate between 75% and 90% in three months follow-up (18,19,24).

High perforation rates documented in this study corroborate with survey reports in Australian aboriginal children and with studies from other developing countries (3,25). The young age at onset combined with the high perforation rate in these rural Bangladeshi children needs to be dealt with cautiously as recurrence, delays in speech, and developmental milestones are common in AOM of early onset in life (10).

Although the number of female subjects who developed the disease was higher than the males in this cohort, the recurrent episodes were higher in males. Results of most epidemiological studies on infectious diseases in infancy showed a male predominance (26).

Findings of research on aetiology of AOM have documented *Streptococcus pneumoniae*, non-typeable *Haemophilus influenzae* (NTHi), and *Moraxella catarrhalis* as important pathogens. Currently, vaccines against *H. influenzae* (NTHi) and *M. catarrhalis* are under development. Pneumococcal conjugate vaccine, licensed in the United States in 2000, was shown in two pivotal trials to reduce the incidence of all causes of AOM by 6%, and pneumococcal AOM by 34%. Pneumococcal AOM caused by serotypes contained in the vaccine was reduced by 57% (27). However, conjugate pneumococcal vaccines are expensive, and the polysaccharide vaccines are not immunogenic before two years of age (28,29). Introduction of such vaccines may be useful in reducing the burden of bacterial AOM. However, research to establish the circulating serotypes of *S. pneumoniae* and *H. influenzae* will be helpful before the introduction of the vaccines. There are three studies indicating that influenza virus vaccines have 30-36% efficacy against the development of AOM. Vaccines to prevent infections with respiratory syncital virus and para influenzae virus type 3 are undergoing clinical testing (27).

The study has several limitations. The findings of AOM were based on the study algorithm which was clinical-based, so culture of ear swabs was not done, hence aetiology remains unknown. Although tympanogram is a useful adjunct for the diagnosis of otitis media in doubtful cases and has a 90% sensitivity and more than 75% specificity to predict middle-ear effusion (30), we have not used this procedure and have relied on the otoscopic findings done by our study physician. However, sophisticated diagnostic procedures are not always feasible in developing countries. Results of field-testing using the WHO algorithm for the Integrated Management of Childhood Illness (IMCI) showed that the sensitivities of ear discharge in diagnosing otitis ranged from 60% to 95%. In Kenya, the sensitivity and specificity of detecting otorrhoea by health workers was 97% and 95% respectively when compared with the physician (31). The long-term outcome of the disease has not been addressed in this study as follow-up was designed until two years of age. We, therefore, suggest that laboratory-based research to identify both bacterial and viral causes of AOM, resistance patterns of organisms causing otitis, and long-term assessment studies in children with history of otitis will be extremely helpful.

In conclusion, this longitudinal follow-up study in children up to two years of age has documented AOM as an important cause of morbidity among the rural children in Bangladesh. The findings of the study will be helpful for policy-makers to bring into focus the burden of the disease. They can plan strategies to reduce morbidity and long-term consequences from this important childhood disease that often goes unnoticed in this country where malnutrition, ARI, and diarrhoea are the main childhood diseases of focus.
